# Biology of the Marine Heterotrophic Dinoflagellate *Oxyrrhis marina*: Current Status and Future Directions

**DOI:** 10.3390/microorganisms1010033

**Published:** 2013-10-21

**Authors:** Zhiling Guo, Huan Zhang, Sheng Liu, Senjie Lin

**Affiliations:** 1 Key Laboratory of Tropical Marine Bio-resources and Ecology, South China Sea Institute of Oceanology, Chinese Academy of Science, Guangzhou 510301, China; E-Mails: zhiling.guo@uconn.edu (Z.G); shliu@scsio.ac.cn (S.L); 2 University of Chinese Academy of Sciences, Beijing 100049, China; 3 Department of Marine Sciences, University of Connecticut, Groton, CT 06340, USA; E-Mail: huan.zhang@uconn.edu; 4 Department of Environmental Science, Ocean University of China, Qingdao 266100, China; 5 Marine Biodiversity and Global Change Research Center, Xiamen University, Xiamen 361005, China

**Keywords:** heterotrophic dinoflagellates, protist, *Oxyrrhismarina*, biology

## Abstract

Heterotrophic dinoflagellates are prevalent protists in marine environments, which play an important role in the carbon cycling and energy flow in the marine planktonic community. *Oxyrrhis marina* (Dinophyceae), a widespread heterotrophic dinoflagellate, is a model species used for a broad range of ecological, biogeographic, and evolutionary studies. Despite the increasing research effort on this species, there lacks a synthesis of the existing data and a coherent picture of this organism. Here we reviewed the literature to provide an overview of what is known regarding the biology of *O*. *marina*, and identify areas where further studies are needed. As an early branch of the dinoflagellate lineage, *O*. *marina* shares similarity with typical dinoflagellates in permanent condensed chromosomes, less abundant nucleosome proteins compared to other eukaryotes, multiple gene copies, the occurrence of *trans*-splicing in nucleus-encoded mRNAs, highly fragmented mitochondrial genome, and disuse of ATG as a start codon for mitochondrial genes. On the other hand, *O. marina* also exhibits some distinct cytological features (e.g., different flagellar structure, absence of girdle and sulcus or pustules, use of intranuclear spindle in mitosis, presence of nuclear plaque, and absence of birefringent periodic banded chromosomal structure) and genetic features (e.g., a single histone-like DNA-associated protein, * cob*-*cox3* gene fusion, 5′ oligo-U cap in the mitochondrial transcripts of protein-coding genes, the absence of mRNA editing, the presence of stop codon in the fused *cob*-*cox3* mRNA produced by post-transcriptional oligoadenylation, and vestigial plastid genes). The best-studied biology of this dinoflagellate is probably the prey and predators types, which include a wide range of organisms. On the other hand, the abundance of this species in the natural waters and its controlling factors, genome organization and gene expression regulation that underlie the unusual cytological and ecological characteristics are among the areas that urgently need study.

## 1. Introduction

Heterotrophic protists are an essential link in the pelagic food webs, as consumers of primary production and prey to upper trophic levels, and can consume more than 60% of daily phytoplankton production from a broad range of oceanic and coastal systems [1,2,3,4]. Heterotrophic dinoflagellates are prevalent in the marine environments, with an abundance of up to 2 × 10^5^ cells·L^−1^ under non-bloom conditions [5,6]. They feed on a broad range of prey species, including phytoplankton, the eggs, early nauplii stages, and adult forms of some metazoans, ciliates, fish bloods and bacteria; at the same time they are important prey for many planktonic consumers, such as some metazoans, ciliates and other dinoflagellates [7,8]. Therefore, heterotrophic dinoflagellates play an important role in the carbon cycling and energy flow in the marine planktonic community [5].

*Oxyrrhis marina* is a widespread, free-living, and ecologically important heterotrophic dinoflagellate [9,10,11]. It is also an important model organism for a broad range of ecological [2,3,12,13,14,15,16], biogeographic [17,18,19], and evolutionary studies [3,10,20]. Despite the increasing number of studies on this organism, the existing data is scattered, remaining to be synthesized. Here we review the biology of this species in hope to provide a coherent picture on this organism and identify areas where further study is needed.

## 2. Taxonomy and Phylogeny of *Oxyrrhis*

Early morphological studies have raised disputes over whether the genus of *Oxyrrhis* contains multiple species (*O*. *marina*, *O*. *maritima* van Meel 1969, *O*. *phaeocysticola* Scherffel 1900 and *O*. *tentaculifera* Conrad 1939) or only one species (*O*. *marina*) [10,21,22,23,24,25]; however, recent molecular phylogenetic studies favor the notion that two sibling species exist in this genus. Cavalier-Smith and Chao [26] suggested that *Oxyrrhis* consists of at least two species based on sequence variations in the small subunit ribosomal RNA gene (SSU rDNA). Lowe *et al.* [27] conducted the phylogenetic analysis of 5.8S rDNA-internal transcribed spacer (5.8S rDNA-ITS) and mitochondrial cytochrome c oxidase I gene (*cox1*) sequences, and found two highly divergent lineages within *O*. *marina* morphospecies, thus proposing the existence of two *Oxyrrhis* species: *O*. *marina* and *O*. *maritima*. A recent phylogenetic analysis of three genes (*cox1*, *α*-*tubulin*, 5.8S rDNA-ITS) for 350 water samples from 149 locations throughout Europe also showed two distinct *O*. *marina* lineages [4]. Since *Oxyrrhis* spp. are ubiquitous in the coastal waters and are easy to be isolated, to date, nearly 400 different *O*. *marina*-like isolates have been reported in the literature [4,18,28,29]; for accurate species identification of the isolates, it is important to use both morphological and molecular methods. For convenience, in this review, we will refer to this species complex as *O*. *marina*.

The phylogenetic position of *O*. *marina* is controversial. Some of the morphological and cytological studies support its basal position in the dinoflagellate lineage [20,30,31], while others infer a highly derived position within the order of Gonyaulacales [24,26,32]. However, with a growing wealth of molecular data on dinoflagellates, such as the phylogenies based on various genes, mitochondrial genome structure, RNA editing, and *trans*-splicing of nucleus-encoded mRNA [32,33,34,35,36], the majority of recent studies supports *O*. *marina* as an ancestral dinoflagellate lineage.

## 3. Unusual Cytological and Genetic Features

*O*. *marina* displays many characteristics that differ from those of typical dinoflagellates. These features make this species cytologically and genetically closer to a typical eukaryote than a typical dinoflagellate.

### 3.1. Morphology

The structure and function of the flagellar apparatus in *O*. *marina* are different from those in other dinoflagellates. The majority of dinoflagellates either have a longitudinal and a transverse flagellum, emerging from the sulcus and the cingulum, respectively, or both flagella growing from the apical area (Prorocentrales). In contrast, *O. marina*’s both flagella grow from the ventral side. Furthermore, the flagella in *O. marina* possess a row of complex mastigonemes while lack a broad striated strand on the transverse flagellum, and both transverse and longitudinal flagella are covered with scales [37,38,39]. In addition, the structure of *O*. *marina* flagellar root system is also significantly different from that of other dinoflagellates, including the breadth of the posteriorly directed microtubular root, the orientation of connective structures and electron dense core of the ventral microtubular root, and the existence of fibers that parallel the flagella [10,40,41].

### 3.2. Nuclear, Mitochondrial and Plastid Genomes

*O*. *marina* shares a few common characteristics with other dinoflagellates as concerns nuclear and organellar genomes. Its nuclear chromosomes remain condensed throughout the cellular cycle [18]; the proteins which constitute the structural basis of the nucleosomes are less abundant than in typical eukaryotic chromatin [42]; *trans*-splicing occurs in nucleus-encoded mRNAs [43,44]; its mitochondrial genome appears to be highly fragmented and mitochondrial genes use non-canonical start codons [34].

*O*. *marina* also exhibits unique nuclear and chromosomal organization which distinguishes it from typical dinokaryotic dinoflagellates [45]. Mitotic cell division of *O*. *marina* is facilitated by an intranuclear spindle rather than an extranuclear spindle observed in typical dinoflagellates [46,47]. In *O*. *marina*, nuclear plaque from which the spindle is generated develops in the nuclear envelope at prophase, but this structure does not appear in the typical dinoflagellates. During division, the nuclear envelope in typical dinoflagellates invaginates to form cytoplasmic channels where the microtubules are present, while in *O*. *marina*, the nuclear envelope does not invaginate and the microtubular mitotic apparatus is intranuclear [31,47]. The birefringent periodic banded or arched chromosomal structure, which is typical in dinoflagellates, has not been observed in *O*. *marina*. While chromosomes divide in the mitosis phase in typical eukaryotes, there is some evidence, which suggests that chromosome division in *O*. *marina* may occur throughout most of the cell cycle [47,48,49]. The movement of the chromosomes in *O*. *marina* is driven by microtubules directly, while in typical dinoflagellates it is driven by microtubules through the nuclear envelope [46,47]. Other differences between *Oxyrrhis* and the typical dinoflagellates include the presence of a large number of long, thin chromosomes that are separated by many electron-dense bodies, the possession of a single histone-like DNA-associated protein, and the absence of a girdle, a sulcus or pustules in *Oxyrrhis* [32].

While dinoflagellates are generally considered to be haploid [50,51], the true ploidy of *O*. *marina* is uncertain [52]. The genome size of a typical dinoflagellate ranges from ~1.5 to over 250 pg·cell^−1^, 0.5–80 times that of a human haploid genome (~3 pg·cell^−1^; [53]). For example, *Symbiodinium* spp. possess relatively small genomes (1.5–4.8 pg·cell^−1^), while *Prorocentrum micans* has a much larger genome, estimated to be 230–280 pg·cell^−1^ [54,55]. There is only one report on *O*. *marina* genome size, with an estimate of 55.8 pg cell^−1^ [48]. This estimate is not consistent with the fact that *O. marina* is cytologically more similar to typical eukaryotes, and therefore remains to be further verified.

To date (June 2013), there are about 19 thousand *Oxyrrhis* nucleotide sequences reported in GenBank databases, most of them are unannotated expression sequence tags (EST); the annotated gene sequence information is mostly restricted to a few genes [e.g., rDNAs, ITS, *actin*, *cox1*, cytochrome *b* (*cob*), rhodopsin, heat shock protein (*hsp*) 90, *α*- and *β*-*tubulin*]. In dinoflagellate genomes, many genes occur in multiple copies (e.g., peridinin-chlorophyll α-binding protein gene (*pcp*) in *Gonyaulax polyedra* by Le *et al.*, 1997 and *Symbiodinium* sp. by Reichman *et al.*, 2003; *actin* in *Amphidinium carterae* by Bachvaroff and Place, 2008; 18S rDNA in *Am*. *carterae*, *Alexandrium taylori* and *Pr*. *minimum* by Galluzzi *et al.*, 2010, *Al*. *fundyense* by Erdner *et al.*, 2010 [56,57,58,59,60]); some genes have been reported to occur in multiple copies in *O*. *marina* (e.g., *actin*, *α*-*tubulin*, *hsp90*, *hsp70* and Adenosylhomocysteinase gene) [49]. The copy number of *actin*, *α*-*tubulin* and *hsp90* in the *O*. *marina* genome was estimated to be 33, 10, 5, respectively [48]. Most of the dinoflagellates genes studied so far are arranged in tandem repeats (e.g., *pcp* in *G*. *polyedra*; luciferase genes (*lcf*) in *Lingulodinium polyedrum*; form II ribulose-1,5-bisphosphate carboxylase gene (*rbcII*) in *Pr*. *minimum*; proliferating cell nuclear antigen gene (*pcna*) in *Pfiesteria piscicida*; *actin* in *Am*. *Carterae*) [56,58,61,62,63,64] although whether they are transcribed as polycistronic transcripts may vary with species and genes (e.g., [61,63,65]). Even in *Perkinsus marinus*, a close alveolate relative of dinoflagellates, some genes such as *cyclin2* occur in tandem repeats [64].

While whole genome sequencing for any dinoflagellate is still impractical due to the excessively large genome size, next generation sequencing can provide efficient strategy to generate functional genomic level data. Recently, *O*. *marina* cultured under different salinity conditions was subjected to transcriptomic analysis using 454 pyrosequencing technology, and about 7000 contigs were retrieved. Bioinformatics analysis of these contigs indicated that some genes occurred as tandem repeats (e.g., *α*- and *β*-*tubulin*, elongation factor 2 gene, rhodopsin, *hsp90*), and multiple transcribed gene variants were common, suggesting a potentially complex genomic arrangements in this organism [66]. In dinoflagellates, some genes are thought to be acquired via lateral (horizontal) gene transfer (LGT) from prokaryotes, e.g., shikimate biosynthesis gene (*aroB*) and *O*-methyltransferase gene (*omt*) from cyanobacteria [67], and *rbcII* from an α-proteobacteria [68,69]. Although LGT has not been examined in detail in *O*. *marina*, it has been reported that *O*. *marina* may have gained genes through LGT via feeding, because the frequent and intimate interaction with prey organisms increase the likelihood of gene transfer [49].

Alveolata, which comprises ciliates, dinoflagellates and apicomplexans [32], has a potentially informative history of mitochondrial genome evolution. Of these lineages, ciliates are thought to branch first from their common ancestor and contain conventional mitochondrial genomes (or mtDNA) [70,71]. For example, ciliates mtDNA is fairly large, and contains 43–52 protein coding genes; its small (SSU) and large (LSU) subunit rRNAs are split into two transcription units. Compared to the large and gene-rich mtDNA in ciliates, the mtDNA of apicomplexans is simple and highly reduced, containing only three protein-coding genes [*cob*, *cox1* and cytochrome oxidase III (*cox3*)] and highly fragmented rRNAs, indicating a considerable mitochondrial gene loss or relocation to the nucleus during alveolate evolution [71]. Despite being sister lineage to apicomplexans and the persistence of some intriguing similarities (e.g., absence of tRNA genes; highly fragmented SSU and LSU rRNAs with similarity in length and sequence termini; the existence of polycistronic transcripts; high level of gene relocation from the mitochondrion; three protein-coding genes), the mtDNA arrangement in dinoflagellates is radically different from that in the apicomplexans, including its complex mitochondrial genome, multiple gene copies in different genomic contexts, RNA editing of protein-coding and rRNA transcripts, non-streamlined mtDNA, loss of stop codons from protein-coding genes, and dispersion of mitochondrial genes over several hundred linear chromosomes [34,71].

As an early branch of the dinoflagellate lineage, *O*. *marina* exhibits some notable exceptions in mitochondrial genome features. *O*. *marina* mtDNA contains the smallest gene complement known to date, with only two protein coding genes and several rRNA fragments [34]. While in most of the dinoflagellate mitochondrial genomes studied so far, *cob*, *cox1* and *cox3* are encoded separately; in *O*. *marina* mitochondria, *cob* and *cox3* are fused to form a single coding unit, and mRNAs are oligo (U) capped at the 5′ end [34,49]. In typical dinoflagellates, no stop codon has been found in *cob*, c*ox1* and *cox3* transcripts, indicating a different translation termination mechanism from other eukaryotes [70,71,72,73]. In *O*. *marina*, although stop codons are absent in *cox1* transcripts, the fused *cob*-*cox3* mRNA seems to have a stop codon that is produced through post-transcriptional oligoadenylation [34]. A similar oligoadenylation mechanism of *cox3* mRNA has also been observed in two other relatively deep-branching dinoflagellates *Am*. *carterae* and *Karlodinium veneficum* [71]. *O*. *marina* mitochondrial genome appears to be duplicated and recombined, as is in other dinoflagellates such as *Am*. *carterae*, *Crypthecodinium cohnii*, *K*. *veneficum*, and *Hematodinium* sp. In addition, mRNA editing of mitochondrial genes, which is common in dinoflagellates, is not found in *O*. *marina* [34,36,74]. This, and similar absence of mRNA editing of mitochondrial genes in other apparently basal dinoflagellate lineages and Apicomplexa have led us to postulate that mitochondrial mRNA editing arose later in dinoflagellate evolution [75]. However, a recent study detected mitochondrial mRNA editing in the parasitic and more basal dinoflagellate *Hematodinium* sp. (in the order of Syndiniales), extending the emergence of mRNA editing to an earlier stage of the dinoflagellate evolution [76], suggesting a possibility that this feature occurred in the common ancestor of dinoflagellates and was subsequently lost in *O. marina* and other basal dinoflagellate lineages.

In non-photosynthetic organisms, photosynthesis-related genes are usually lost or have become pseudogenes [74]. In *O*. *marina*, however, a number of genes encoding proteins associated with the plastid or photosynthesis have been identified (e.g., ketol-acid reductoisomerase, carbonic anhydrase, cysteine synthase, 1-deoxy-d-xylulose-5-phosphate reductoisomerase, haem, ascorbate peroxidase, glutamine synthetase, hydroxymethy lbilane synthetase, ribulose 5-P isomerase, and dihydrodipicolinate reductase) [9,66]. Moreover, plastid-targeting peptides have been detected in some proteins of this species. All these suggest that *O*. *marina* may have evolved from a plastid-bearing ancestor and a cryptic plastid may still exist in *O*. *marina* to fulfill some metabolic pathways [9]. Vestigial plastid genes have also been found in some parasitic and heterotrophic alveolates such as the Apicomplexa [77,78,79], ciliates [80,81], and *Perkinsus* [82,83]. It is reported that the relict plastid can support essential metabolic functions in Apicomplexa, including haem biosynthesis, type II fatty acids synthesis, and isoprenoid precursor biosynthesis, which play an important role for parasite survival in different host settings [84]. These results are consistent with the suggestion that chromavelolates share a common photosynthetic ancestor [85,86,87]. Photosynthesis-related gene *rbcII* has been found in the non-photosynthetic dinoflagellate *C*. *cohnii* [74]; whether this gene is present in *O*. *marina* is unclear but would be worth investigating as it can provide valuable insights into the evolution of *rbcII* in dinoflagellates.

## 4. Nutritional Modes

*O*. *marina* represents an intriguing mix of feeding strategies compared with most heterotrophic protists. This includes phagotrophy, saprotrophy, cannibalism, and phototrophy (harvesting solar energy to enhance growth).

### 4.1. Phagotrophy

**Figure 1:**
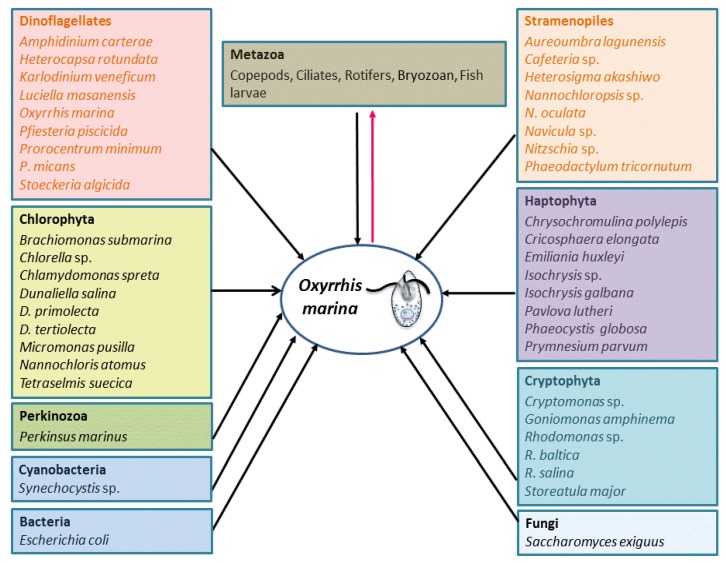
The schematic showing relationships of *O*. *marina* with other organisms as prey and predators as documented in literature. The black-colored arrows emerging from the variety of prey all point to *O*. *marina* as the grazer, while the red-colored arrow emerging from *O*. *marina* leads to the diverse predators of *O*. *marina*.

*O*. *marina* can feed by phagotrophy on a variety of prey belonging to diverse taxa and size categories, including phytoplankton, the eggs and nauplii of metazoa, ciliates and bacteria (Figure 1; [9]). The typical cell size of *O*. *marina* is 20–30 μm [18]. Its optimum prey size is smaller than its own size, and it grows at high rates when feeding on small flagellates (size ≥ 4 μm) [88,89,90,91]. It ingests prey by engulfment, which appears to be more efficient than other feeding mechanisms [5]. Maximum ingestion and clearance rates of *O*. *marina* on phytoplankton range from 0.07 to 2.8 ng C day^−1^ per grazer and 0.002–0.015 μL per grazer·h^−1^, respectively [2,5,88]. Jeong *et al.* [8] reported that bacteria alone could support the growth of *O*. *marina*, and that the ingestion and clearance rates on bacteria could be up to 71.3 and 31.3 cells·h^−1^, respectively, much higher than those of other heterotrophic dinoflagellates.

The maximum growth rate of *O*. *marina* is relatively high, ~0.033 h^−1^ when fed with *Dunaliella tertiolecta* or *Isochrysis galbana*, and 0.054 h^−1^ with *Phaeodactylum tricornutum* [5,88]. Although *O*. *marina* demonstrates remarkable versatility in its prey, it has feeding preferences. When fed with artificial particle prey, *O*. *marina* exhibits selective feeding based on the size, biochemical composition and charge of the particles. For example, *O*. *marina* prefers 4 μm over 1 μm beads, and beads coated with mannose-BSA over *N*-acetylgalactosamine-BSA [92,93,94].

Even though *O*. *marina* can ingest artificial particles, it prefers live prey and can discriminate between different species. It has been reported that *O*. *marina* can use the mannose-binding lectin on the cell membrane to distinguish different prey species [94]. A number of studies have investigated the prey selectivity of *O*. *marina*, yet because they used different initial ratios of prey and predator, biomasses and biovolumes, incubation conditions and durations, their conclusions were different [88,89,95,96,97]. In addition, many traits of prey, such as cell size and shape, motility, hydrophobicity, nutritional value, dissolved chemical cues, and cell surface properties, can vary between different prey species. Therefore, it is difficult to determine the factors that influence the prey selectivity between species [5,98,99,100,101,102]. *O*. *marina* can also discriminate individuals of the same species with different properties. For example, it grazes selectively on virus-infected over healthy *Emiliania huxleyi*, and low dimethylsulphoniopropionate (DMSP) lyase-activity strains over high DMSP lyase-activity strains [103,104].

### 4.2. Saprotrophy

In addition to phagotrophy, *O*. *marina* can survive through the uptake of dissolved organic molecules, indicating its capability to feed by saprotrophy. When cultured in f/2 medium supplied with ethanol or acetate as carbon source but free of complex nutrients, *O*. *marina* still grew successfully [105]. It has been reported that *O*. *marina* is able to synthesize the full complement of amino-acids by using ammonium or other nitrogen sources as substrate [106,107]. Slamovits and Keeling [9] reported that *O*. *marina* has Ketol-acid reductoisomerase, which is involved in the synthesis of the essential amino acids Valine, Leucine and Isoleucine. By analyzing a 454-based transcriptome dataset obtained from *O*. *marina*, Lowe *et al.* [66] identified a broad range of genes associated with amino acid synthesis and metabolism, indicating the biosynthetic capacities of this species.

### 4.3. Cannibalization

*O*. *marina* is known to be cannibalistic in a food-deficient environment, but usually only a small proportion (~2%) is cannibals in a population. Öpik and Flynn [108] found ingested *Oxyrrhis* cells in *O*. *marina* by electron microscopic observation. Factors that drive feeding on conspecific cells are not well understood, but cannibalism may help the population to survive when their usual prey is scarce [3,5].

### 4.4. Phototaxis, Circadian Rhythm, and the Potential of Solar Energy Utilization

As in other unicellular organisms, photosensory capability exists in many heterotrophic protists [109,110,111,112,113,114,115]. Hartz *et al.* [19] observed that *O*. *marina* exhibited positive phototaxis to different wavelengths of light, suggesting its ability to use photosensory response to orient in their environment and locate prey.

*O*. *marina* seems to possess an endogenous circadian clock that controls its feeding and growth. Jakobsen and Strom [116] examined the responses of feeding (and growth) rates in *O*. *marina* to both the light: dark cycle and continuous darkness treatments, and found that *O*. *marina* fed and divided with a diel rhythm, with higher feeding and growth rates during the light period, and the persistence of diel differences in feeding and growth during 24-h exposure to continuous darkness. These results demonstrated that the circadian rhythm was controlled by biochemical processes endogenously generated. They also observed that *O*. *marina* lost its diel cycle when removing the external modulating light cue for 230 h, indicating that the circadian rhythm was cued by a light-modulated signal, and the irradiance threshold for maintenance of the circadian cycle was 3.1 × 10^−4^ μmol photons m^−2^·s^−1^.

*O. marina* exhibits some phototrophic capabilities. It has been reported that light may stimulate grazing and enhance population growth of mixotrophic and heterotrophic protists [117,118,119,120]. The light-dependent benefit may come from phototaxis, as observed in *O*. *marina*, which may motivate the prey searching activity [2,116], but it may also come from photodegradation of ingested food and stimulation of digestive enzymes as postulated for other protists [117,121]. It has been observed that growth and feeding rates of *O*. *marina* is higher in the light than in the dark [2,116]. Klein *et al.* [122] found that light promoted alloxanthin and chlorophyll c degradation by *O*. *marina*. Besides, Lin and colleagues found that *O*. *marina* and other dinoflagellates possessed and expressed a proton-pump type rhodopsin, which was highly similar to proteorhodopsin previously found in a wide range of proteobacteria living in the ocean surface [42,123,124,125]. Whether dinoflagellate rhodopsin has been acquired through horizontal gene transfer from marine bacteria remains to be further studied.

Although the function of *O. marina* rhodopsin is still unclear, it is suspected that this type of rhodopsin may generate energy from light directly to power biological functions, or acidify digestive vacuoles using light rather than ATP [126]. Furthermore, by sequencing about 18,000 EST from *O*. *marina*, Slamovits *et al.* [126] found that there were two different types of rhodopsin, and proposed that one of them was sensory rhodopsin. Different types of rhodopsin may express differentially and play different physiological roles.

## 5. Swimming and Feeding Behaviors

### 5.1. Swimming Behavior

Early studies showed that the two morphologically and functionally differentiated flagella were responsible for the swimming motion of *O*. *marina* [127,128]. One of the flagella is the extending longitudinal flagellum which produces the planar and symmetrical waves to push the cell forward; the other is the coiled transverse flagellum which partially wraps around the cell from its origin in a ventral furrow and produces the helical waves to propel the cell forward [39,127,128]. The beating of these two flagella causes *O*. *marina* to swim in three-dimensional helical path, generally with a 44–80 μm helical width, and a mean forward displacement of 400–700 μm·s^−1^ for completing a full helical rotation [129]. The helical swimming-related parameters, including the swimming speed, rotational movement, helical extent and directional persistence, can be affected by environmental stimuli (e.g., food items and chemical cues) [16,130]. Since the volume of water sampled across the net direction of movement is wider and the searching radius is increased when swimming in a helix, the foraging efficiency of this organism is higher [131]. *O*. *marina* also exhibits a run-and-tumble swimming behavior, where it reorients itself by folding the longitudinal flagellum after periods of long-straight movements [16,129].

Swimming behavior is associated with the abundance of the prey. It has been recorded that more frequent turning, shorter runs and higher swimming speed occur in the presence of food than when without food [132,133,134]. Swimming behavior of *O*. *marina* also varies with different prey concentrations, and these changes are physically controlled by the flagellar movement, involving more energy in using longitudinal flagellum at high prey concentration (~10^4^–10^5^ cells·mL^−1^) and performing frequent transverse flagellum movement at low prey concentrations (~10^1^–10^3^ cells·mL^−1^) [2]. *O*. *marina* is capable of directed swimming which contributes to exploiting patchily distributed resources [16,134]. Swimming speed, changes in the direction, and frequency of turning will subsequently influence spatial distribution and the prey searching of *O*. *marina* [2]. For example, in the presence of food, higher swimming speed and turning angles result in a decrease in the diffusivity of *O*. *marina* [134].

### 5.2. Feeding Behavior

*O*. *marina* feeding includes several stages: prey searching, contacting and capturing, processing, ingesting and digesting [2]. Prey searching is triggered by the environmental stimulus and usually is a process of the recognition and detection of the dissolved chemoattractants by the predator. Generally *O*. *marina* exhibits a positive motile response in the presence of various chemical cues, including prey cells, prey exudates and infochemicals; this response involves cell signal transduction process which is initiated by the binding of a chemical molecule released by prey to a specific cell surface receptor of *O*. *marina* [2,134]. It has been reported that G-proteins, G-proteins-coupled receptors, and protein kinases may be involved in the signaling pathways initiating motile behavior [135].

The detailed mechanisms underlying contacting, capturing, and processing prey in this organism are still poorly understood, because its swift movement and rapid engulfment of prey make it challenging to observe accurately. Fenchel [136] reported that *O*. *marina* exhibited two different feeding behaviors: raptorial feeding and filter feeding, depending on the size of prey. Jeong *et al.* [8] investigated the feeding behavior of *O*. *marina* on marine bacteria, and found that the bacteria were trapped in the feeding currents produced by the transverse flagellum of *O*. *marina* and were moved toward the cingular depression, where the bacteria were engulfed, indicating that this species is an intercept feeder of bacteria. *O*. *marina* shows more active and vigorous performance when the prey is a protist. It has been reported that within 15 min of addition of *Dunaliella*, up to three prey cells could be seen inside individual *Oxyrrhis*, with 10 and 15–20 prey cells by 3 h and 9 h, respectively [108]. Therefore, *O*. *marina* may be advantaged in gaining energy by using different feeding behaviors (raptorial, filter and interception feeding).

There are contradictions regarding the time of contact of prey and predator and the involvement of trichocysts in prey capture. For example, the contact of prey and predator was noted to occur prior to [137] or following [8,138] the encircling of prey by *O*. *marina*. In some cases the trichocysts and filaments were observed to involve in the prey capture activity [8,108]; however, studies by Höhfeld and Melkonian [137] suggested the absence of trichocysts during the stages of phagocytosis.

The prey processing is a stage during which *O*. *marina* may select the prey and decide whether or not to accept certain food types [101,139]. Following this is the ingestion phase by phagocytosis, which involves the rearrangement of the microtubular cytoskeleton. During this stage, prey is firstly adhered to the predator through the binding of receptors on predator to ligands on the cell surface of prey, and then ingested by phagocytosis in a temporary cytostome through protein kinase-based signaling pathways [2,135]. After the prey is engulfed, the cells firstly become more electron-dense and the contents coagulated, and later less distinct and disintegrated to granular-membranous masses, and finally only the vesicular deposits are identifiable and the prey is completely digested [108].

Being an important predator on the one hand, *O*. *marina* is a suitable food source and an appropriate prey size for a range of organisms on the other hand. *O. marina* predators include fish larvae, copepods (*Acartia clause*, *Temora longicornis* and *Centropages hamatus*), ciliates, and rotifers (Figure 1; [3,140,141]). For examples, *O*. *marina* fed with *Dunaliella* sp. synthesizes long-chain fatty acids and sterols; using this *O*. *marina* culture as food for copepods results in higher growth rate than copepods only supplied with *Dunaliella* [142]. *O*. *marina* can provide rotifer predators with tryptophan that is not abundant in the algal prey [143]. There is report that *O*. *marina* sometimes could become food of its prey when the nutrient is stressed. For example, mixotrophic haptophyte *Prymnesium parvum* is a suitable prey for *O*. *marina* under normal conditions, but when exposed to nutrient-stressed *P*. *parvum* cultures, which produce toxin, *O*. *marina* loses its motility, lyses and finally is consumed by *P*. *parvum* [12,13].

## 6. Life Cycle and Cell Cycle

### 6.1. Life Cycle

Most of the dinoflagellates have a haplontic life cycle [50,51]. The life cycle of dinoflagellates is influenced by the ambient environmental conditions. Under favored conditions, cells divide and the population grows by binary fission, while in stressed conditions, cells act as gametes and fuse into a diploid zygote which then undergoes a meiotic division to produce new haploid cells [144]. So far, little is known about the life cycle of *O*. *marina*. It is believed that *O*. *marina* is isogamous; gametic cells are formed by two typical meiotic divisions and consequently are much smaller than vegetative cells [18]. Montagnes *et al.* [18] observed small *O*. *marina* cells (~8 μm) from all of the ~400 isolates of *O*. *marina* cultures, and proposed that they might be gametes; however, there was no evidence of fusion of these mini-cells.

### 6.2. Cell Cycle

In *O*. *marina*, cell division is by transverse fission. As in other dinoflagellates, the chromosomes are always condensed during the cell cycle, but the structural organization of division is different from other dinoflagellates [18]. Similar to the typical eukaryotes, the cell cycle of the exponentially growing *O*. *marina* cells contains four phases: G_1_, S, G_2_, and M, with the major portion (50%) being in G_2_ and M phases [48,145], the two phases that are indistinguishable by flow cytometry, the method commonly used to analyze the cell cycle. Cells in stationary phase usually accumulate in G_1_ and G_2_, and nutrient-stressed cells accumulate at G_2_ to allow rapid exploitation of new resources once the nutrients are replete [145], suggesting the nutrient-dependent cell cycle control points are located in both of these phases to allow *O*. *marina* to adapt to the feast-or-famine conditions. *P*. *piscicida*, another heterogrophic dinoflagellate, also tends to accumulate in G2 when prey is depleted, apparently to allow rapid population recovery by entering cell division once prey is resupplied [146].

## 7. Physiology

A well-fed, dense *O*. *marina* culture has pinkish color [20,147]. We observed the same phenomenon, but usually after prey have been consumed, suggesting induction of rhodopsin as a result of accelerated food digestion or food depletion. The cell size and shape are highly variable in *O*. *marina* due to different osmotic conditions, temperature, food concentrations and culture status, and can also be clone-specific [10,148]. Jonsson [149] found that in intertidal rock pools *Oxyrrhis* sp. formed robust adherent cysts associated with the tidal cycle. Recently Montagnes *et al.* [18] also found thin-membrane covered cysts in *O*. *marina* cultures. Resting cysts have also been described in other dinoflagellates, with functions ranging from survival during adverse conditions, bloom initiation and termination, and a seed bank for genetic diversity and wide dispersal [150,151,152,153].

The growth rate of *O*. *marina* is affected by various abiotic factors, including salinity [17,154], turbulence [155], temperature [148], pH [156] and tidal cycle and height [149]. *O*. *marina* can grow at 0.67 and 0.34 day^−1^, respectively, when cultured in 30 and 50 practical salinity unit (PSU) Droop’s S69 axenic growth medium [66]. High turbulence has negative effect on *O*. *marina* growth, which can decrease growth rate by ~20% [155], while low-level turbulence can increase the encounter with prey, and hence increase the growth [157]. Jonsson [149] investigated the relationship between *O*. *marina* abundance and tidal action, and identified a positive correlation between tidal height and abundance of *O*. *marina* on the Isle of Man. *O*. *marina* exhibits strain-specific differences of physiological responses to a diversity of environmental factors [158].

## 8. Geographic Distribution

Firstly isolated from a salt marsh in Belgium over 150 years ago, *O*. *marina* is commonly found in shallow waters and littoral and supralittoral pools, but rarely found in the open oceans [3,11,143,148,159]. It is globally distributed in coastal and intertidal waters, having been reported in the Atlantic and Pacific coasts of the USA, the Gulf of Mexico, the Atlantic coasts of Europe, the Mediterranean sea, the Persian Gulf, the Indian Ocean and the western Pacific [11], the Gulf of Finland and the south and west Baltic and Red Seas [160], the White Seas [24], intertidal pools on the Isle of Man [159], the Amursky Bay in Japan [147,161], the Southern Bay of Biscay [162], and the Chesapeake Bay [163]. Undoubtedly, the wide distribution of *O*. *marina* is associated with its eurytopic characteristics and its ability to tolerate varying environmental conditions.

Lowe *et al.* [27] conducted phylogeographic analyses using *cox1* and 5.8S rDNA-ITS on 58 *Oxyrrhis* isolates from a range of locations and reported that *O*. *marina* showed high genetic diversity and broad-scale spatial structure. Two highly divergent lineages existed within *O*. *marina* morphospecies, with each composed of two distinct clades, with clades 1 and 2 (lineage 1) more abundant and widespread, and clades 3 and 4 (lineage 2) rare and spatially restricted [27]. Furthermore, using highly polymorphic microsatellites markers, Lowe and colleagues found that *O*. *marina* had high genetic diversity even on small spatial scales [28]. The high genetic diversity may reflect adaptive variation in this species. It is reported that *O*. *marina* can tolerate a wide range of temperature (8–30 °C), salinity (4–130 PSU), and pH (7.8–9.8) [11,18,65].

Although there are extensive reports on the geographic distribution of *O*. *marina*, information on its abundance is rare. *O*. *marina* was observed to form a bloom in Amursky Bay, in the coastal water of the Northern Sea of Japan in July 2002, with the maximum cell concentration of 4.4 × 10^5^ cells mL^−1^ [147]. Johnson *et al.* [163] noted the abundance of *O*. *marina* at about 0–100 cells·mL^−1^ in summer in Chesapeake Bay, MD, USA. All the quantitative data of *O*. *marina* abundance currently available were based on microscopic identification, which is time-consuming and technically challenging because the samples may have distorted cell morphology, making *O*. *marina* species identification difficult microscopically [164,165,166]. Over the past decades, several taxon-specific molecular detection approaches based on signature of genetic variability have been developed, among which real-time quantitative PCR (qPCR) has been successfully used to quantify many dinoflagellate species including *Al*. *fundyense* [167], *Al*. *minutum* [166], *Al*. *tamarense* [168] (likely *Al. fundyense* sensu Wang *et al.* 2013 [169]), *Al*. *catenella* and *Al*. *taylori* [59], *Karenia mikimotoi* [170], *K. veneficum* [171], *Pf*. *piscicida* [172], *Pf*. *shumwayae* [165], and *Symbiodinium* sp. [173]. Therefore, molecular techniques such as qPCR are needed for rapid detection and quantification of *O*. *marina* from environmental samples in the future, and information on *O*. *marina* abundance in the marine ecosystem is needed to better understand the seasonal- and long-term population dynamics and thus ecological roles of this species. Furthermore, the mechanisms that sustain the high levels of genetic diversity in this organism need to be uncovered.

## 9. Future Directions for Research on *O*. *marina*

**Figure 2:**
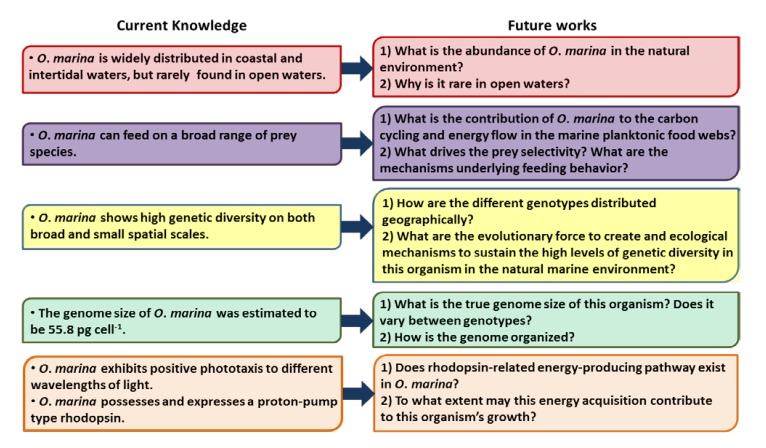
Diagram summarizing the current status of knowledge in key areas and showing proposed future research topics to address the inadequacy of those areas.

As a phylogenetically basal lineage and a widely distributed species of the heterotrophic dinoflagellates, *O*. *marina* represents an excellent model for evolutionary and ecological studies of dinoflagellates. Despite the increasing attention paid to this species, there are still many unanswered questions (Figure 2). For example, what is the abundance of *O*. *marina* in the natural environment? How are the different genotypes distributed geographically? What are the evolutionary force to create and ecological mechanisms to sustain the high levels of genetic diversity in this organism in the natural marine environments? Does the wide range of prey species *O*. *marina* is able to consume in the laboratory reflect a broad prey spectrum of this species in the natural environments and does this flexibility “buffer/dampen” predator-prey cycles? What is the genome size of this organism and how is the genome organized? Does rhodopsin-related energy-producing pathway exist in *O*. *marina*, and if so, to what extent may this energy acquisition contribute to this organism’s growth? Based on these questions, below we will propose some ideas for future studies.

### 9.1. *O. marina* Abundance in the Natural Environment

Quantifying the abundance of *O*. *marina* in the natural environment may gain insights into the seasonal- and long-term population dynamics and thus ecological roles of this species. *O*. *marina* is a non-thecate dinoflagellate; samples preserved may have distorted cell morphology, making *O*. *marina* species identification difficult under the microscope. Molecular techniques, especially qPCR methods are needed to quantify the abundance of *O*. *marina*. The accuracy of the qPCR technique to quantify phytoplankton in the natural environments depends on the efficient isolation of high quality DNA quantitatively from water samples. To verify that the DNA extraction method is highly efficient, a control procedure is needed. Firstly, the DNA content of *O*. *marina* needs to be measured accurately. Among other methods, flow cytometry, a method commonly used to measure the genome size of different organims is an option [54,55]. Different sample collection, preservation, and DNA extraction methods are compared and the one which gives the closest DNA content with that measured by flow cytometry is chosen for future DNA extraction and the DNA recovery efficiency is calibrated.

18S rDNA is widely used in qPCR because it consists of multiple copies in the genomes of eukaryote and hence a highly sensitive gene marker [165]. The number of 18S rDNA copies in *O*. *marina* needs to be measured in order to accurately convert copies per sample to cells per sample. The abundance of *O*. *marina* in the natural samples can then be quantified using the qPCR method established.

### 9.2. *O. marina* Transcriptomic Study

While it is still not feasible to completely sequence *O. marina* genome, information on genome structure and function can be gained through gene expression profiling (transcriptomic analysis). Since *O. marina* is a heterotrophic dinoflagellate, a technical issue associated with the transcriptomic studies is the contamination of the prey cells. The recent discovery of the spliced leader (SL) *trans*-splicing in dinoflagellate mRNAs offers a unique tool to separate dinoflagellate transcripts from assemblage of different organisms [42,43]. The cDNA libraries of *O. marina* cultured in different conditions can be constructed using a PCR-based method with DinoSL as a forward primer and sequenced using next generation sequencing method. After the functional annotation, the differential expression gene can be retrieved and the interested genes (such as food-related, growth-related genes) can be isolated for future studies.
